# Author Correction: TRPV1 SUMOylation regulates nociceptive signaling in models of inflammatory pain

**DOI:** 10.1038/s41467-018-05022-w

**Published:** 2018-06-28

**Authors:** Yan Wang, Yingwei Gao, Quan Tian, Qi Deng, Yangbo Wang, Tian Zhou, Qiang Liu, Kaidi Mei, Yingping Wang, Huiqing Liu, Ruining Ma, Yuqiang Ding, Weifang Rong, Jinke Cheng, Jing Yao, Tian-Le Xu, Michael X. Zhu, Yong Li

**Affiliations:** 1 Department of Biochemistry and Molecular Cell Biology, Shanghai Key Laboratory for Tumor Microenvironment and Inflammation, Institute of Medical Sciences, Shanghai Jiao Tong University School of Medicine, Shanghai, 200025 China; 2 Hubei Key Laboratory of Cell Homeostasis, College of Life Sciences, Wuhan University, Wuhan, Hubei 430072 China; 3 Department of Anatomy and Neurobiology, Collaborative Innovation Center for Brain Science, Tongji University School of Medicine, 200092 Shanghai, China; 4 Department of Anatomy and Physiology, Shanghai Jiao Tong University School of Medicine, 200025 Shanghai, China; 5 Department of Integrative Biology and Pharmacology, McGovern Medical School, The University of Texas Health Science Center at Houston, Houston, TX 77030 USA

Correction to: *Nature Communications* 10.1038/s41467-018-03974-7, published online 18 April 2018

In the originally published version of this Article, the affiliation details for Yan Wang, Yingwei Gao, Qi Deng, Yangbo Wang, Tian Zhou, Yingping Wang, Huiqing Liu, Ruining Ma, Jinke Cheng and Yong Li incorrectly omitted ‘Shanghai Jiao Tong University’. This has now been corrected in both the PDF and HTML versions of the Article.’

Furthermore, the Supplementary Information file originally associated with this Article inadvertently omitted Supplementary Figure 9. The error has now been fixed and the corrected version Supplementary Information PDF is available to download from the HTML version of the Article.

