# Supplementary material for: TRPV1 SUMOylation regulates nociceptive signaling in models of inflammatory pain
Source: Nat Commun. 2018 Apr 18;9:1529. doi: 10.1038/s41467-018-03974-7 (PMC5906468; doi:10.1038/s41467-018-03974-7)

**Supplementary Figures**


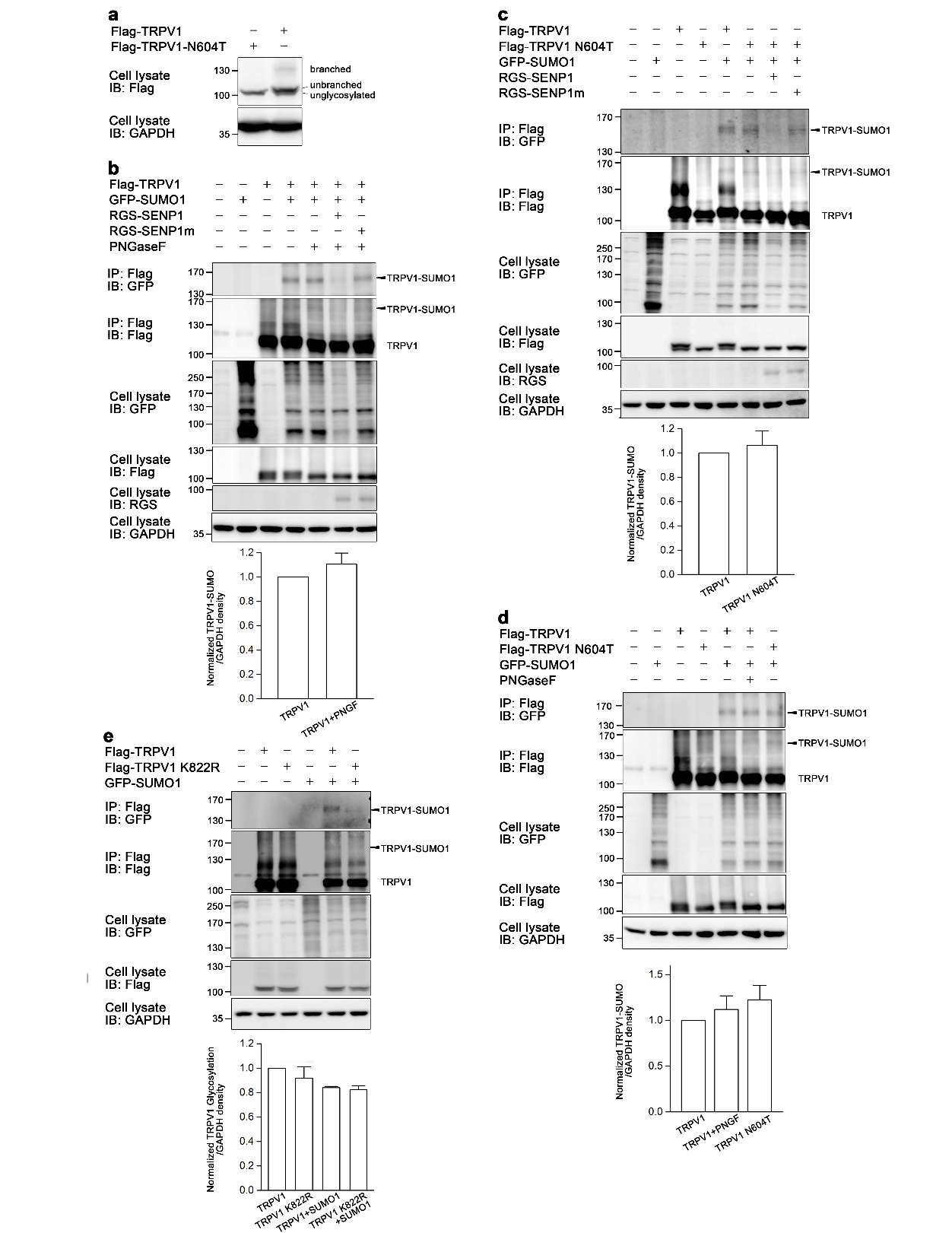


**Supplementary Figure 1. TRPV1 SUMOylation is not dependent on glycosylation.** (**a**) CHO-K1 cells were transiently transfected with Flag-TRPV1 or Flag-TRPV1-N604T (Non-glycosylation mutant). Cell lysates were analyzed by IB with Flag antibody. (**b**) Cells were transiently co-transfected with Flag-TRPV1, GFP-SUMO1, RGS-SENP1 and RGS-SENP1m. Cell lysates were incubated with PNGase F for 1 h at 37 °C, then IP with anti-Flag antibody and analyzed by IB using anti-GFP or anti-Flag. (**c**) Cells were transiently co-transfected with Flag-TRPV1, Flag-TRPV1-N604T, GFP-SUMO1, RGS-SENP1 and RGS-SENP1m. Cell lysates were IP with anti-Flag antibody and analyzed by IB using anti-GFP or anti-Flag. (d) Cells were transiently co-transfected with GFP-SUMO1 and Flag-TRPV1 or Flag-TRPV1-N604T. Cell lysates were incubated with PNGase F as in (**b**), then IP with anti-Flag antibody and analyzed by IB using anti-GFP or anti-Flag. (**e**) Cells were transiently co-transfected with GFP-SUMO1 and Flag-TRPV1 or Flag-TRPV1-K822R. Cell lysates were IP with anti-Flag antibody and analyzed by IB using anti-Flag. Data are means ± s.e.m from three independent experiments. Student’s *t*-test for **b**, **c**, **d** and **e**.


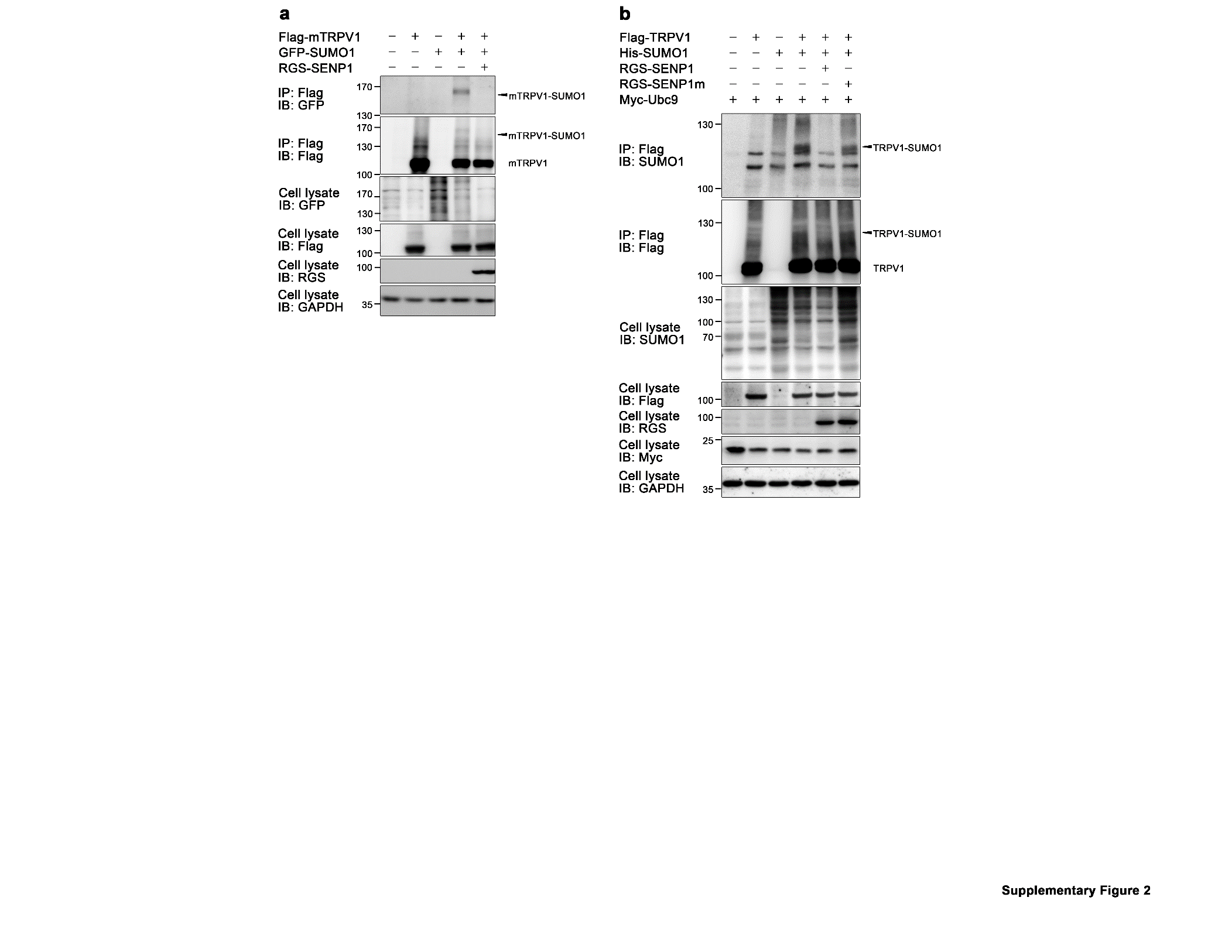


**Supplementary Figure 2. SUMO1 modification of TRPV1.** (**a**) Conjugation of mouse TRPV1 by GFP-SUMO1 co-expressed in CHO-K1 cells. Flag-mouse TRPV1, GFP-SUMO1 or RGS-SENP1 were transfected into CHO-K1 cells as indicated: Cell lysates were subjected to IP by anti-Flag, which was followed by IB for GFP and Flag. (**b**) Conjugation of rat TRPV1 by His-SUMO1 co-expressed in HEK293T cells. Cells were transiently co-transfected with Flag-TRPV1, His-SUMO1, Myc-Ubc9, RGS-SENP1 and RGS-SENP1m. Cell lysates were IP with anti-Flag antibody and analyzed by IB using anti-TRPV1 or anti-SUMO1. Data presented in this Supplementary Figure 2, a and b have been reproduced at least three times.


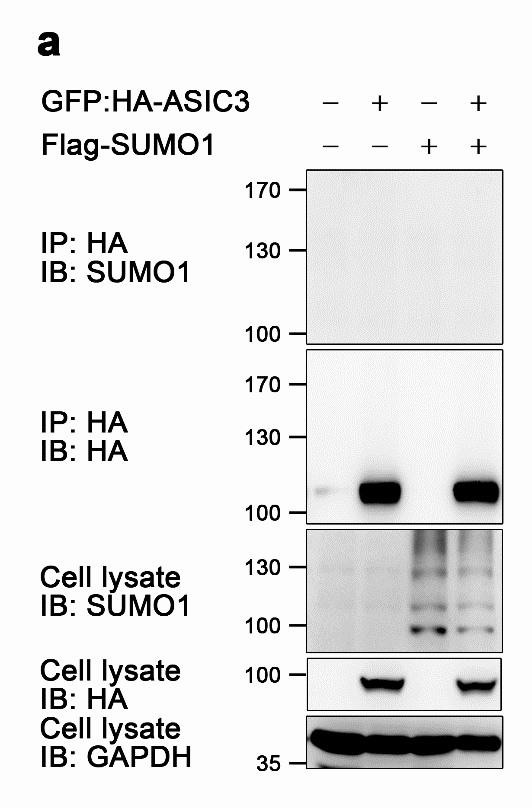


**Supplementary Figure 3. ASIC3 is not SUMOylated by SUMO1.** (**a**) Lysates from CHO-K1 cells transfected with GFP:HA-ASIC3 or co-transfected with GFP:HA-ASIC3 and Flag-SUMO1 were IP with anti-HA antibody and analyzed by IB using anti-SUMO1 and anti-HA antibodies. No SUMO1 signal was detected in the IP HA. Whole-cell lysates were used for IB with anti-SUMO1, anti-HA and anti-GAPDH antibodies for input. Data shown in this Supplementary Figure 3 are representative of at least three independent experiments.


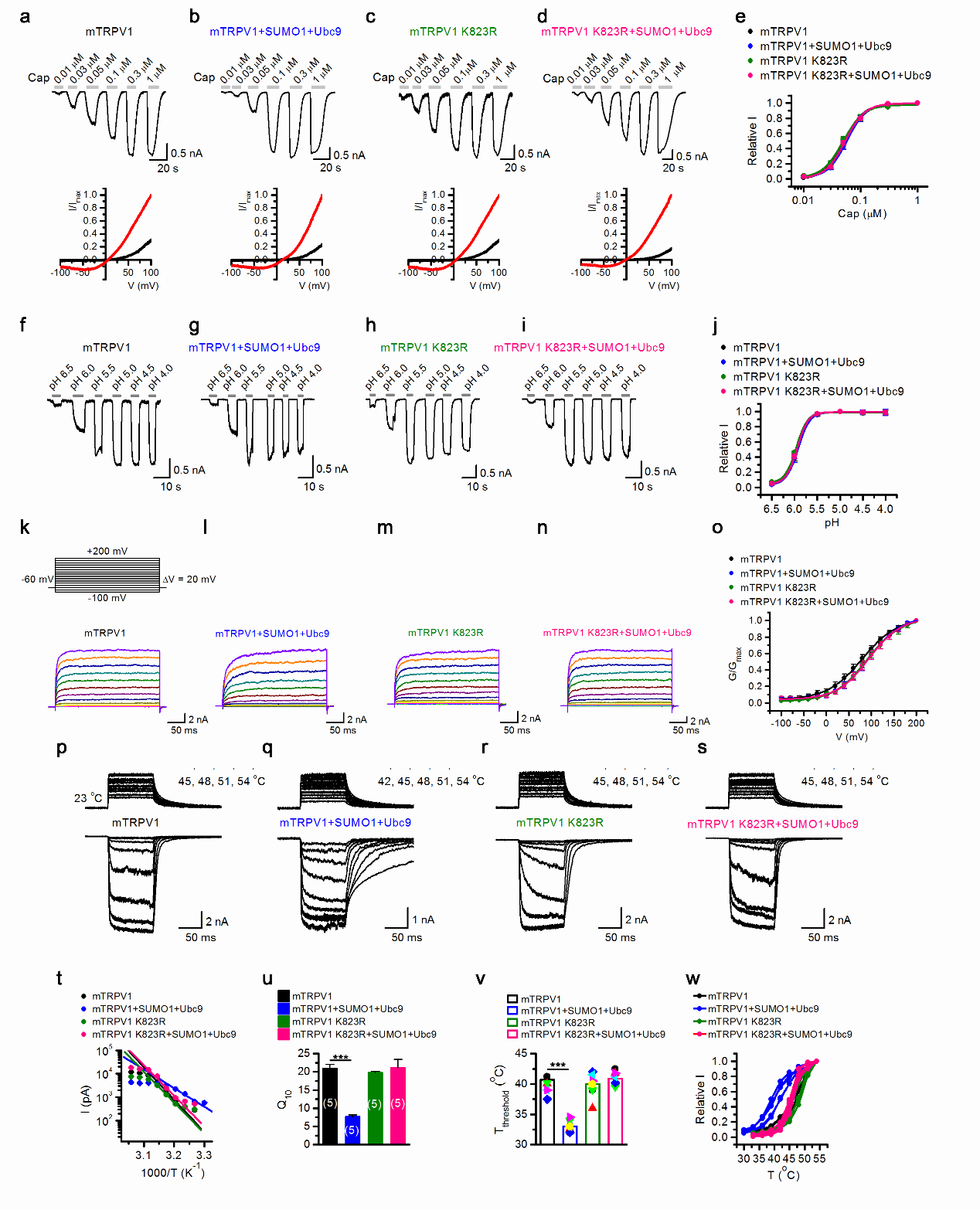


**Supplementary Figure 4. SUMOylation lowered the temperature threshold (T_threshold_) of mouse TRPV1 activation in CHO-K1 cells.** (**a-d**) Upper graphs are representative traces for currents at -60 mV evoked by increasing concentrations of capsaicin as indicated for CHO-K1 cells that expressed Flag-mTRPV1 (**a**), Flag-mTRPV1 + GFP-SUMO1 + Ubc9 (**b**), Flag-mTRPV1-K823R (**c**) and Flag-mTRPV1-K823R + GFP-SUMO1 + Ubc9 (**d**). Lower graphs are the corresponding I-V relationships of TRPV1 in response to Voltage ramp from -100 mV to +100 mV alone (*black*), or combined with 0.03 μM Cap (*red*). (**e**) Concentration-response curves for capsaicin-evoked currents. Solid lines indicate fits with the Hill equation, which yielded the following result: for mTRPV1-WT (*black*), EC_50_ = 50.9 ± 3.1 nM, and n_H_ = 2.5 ± 0.3 (n = 12); for mTRPV1-WT+SUMO1+Ubc9 (*blue*), EC_50_ = 56.9 ± 2.2 nM, and n_H_ = 2.6 ± 0.2 (n = 7); mTRPV1-K823R (*olive*), EC_50_ = 49.3 ± 3.5 nM, and n_H_ = 2.4 ± 0.4 (n = 7); for mTRPV1-K823R+SUMO1+Ubc9 (*pink*), EC_50_ = 54.2 ± 3.4 nM, and n_H_ = 2.5 ± 0.3 (n = 7). Data points are means ± s.e.m. (**f-i**) Representative traces for currents at -60 mV evoked by solutions with decreasing pH values as indicated for CHO-K1 cells that expressed Flag-mTRPV1 (**f**), Flag-mTRPV1 + GFP-SUMO1+Ubc9 (**g**), Flag-mTRPV1-K823R (**h**) and Flag-mTRPV1-K823R + GFP-SUMO1+Ubc9 (**i**). (**j**) Concentration-response curves for proton-evoked currents. Best fitting with Hill equation yielded the following results: for mTRPV1 (*black*), pH_0.5_ = 5.9 ± 0.1, and n_H_ = 3.3 ± 0.2 (n = 11); for mTRPV1-WT+SUMO1+Ubc9 (*blue*), pH_0.5_ = 5.9 ± 0.1, and n_H_ = 3.4 ± 0.2 (n = 6); for mTRPV1-K823R (*olive*), pH_0.5_ = 6.0 ± 0.1, and n_H_ = 3.6 ± 0.5 (n = 6); for mTRPV1-K823R+SUMO1+Ubc9 (*pink*), pH_0.5_ = 5.9 ± 0.1, and n_H_ = 3.6 ± 0.2 (n = 6). Data points are means ± s.e.m. (**k-n**) Representative traces of currents evoked by a family of voltage steps from -100 to +200 mV with 20 mV increments as shown in inset in (**k**) for CHO-K1 cells that expressed Flag-mTRPV1 (**k**), Flag-mTRPV1 + GFP-SUMO1 + Ubc9 (**l**), Flag-mTRPV1-K823R (**m**) and Flag-mTRPV1-K823R+ GFP-SUMO1 + Ubc9 (**n**). Holding potential was -60 mV. (**o**) Conductance-voltage (G-V) relationships derived from the experiments shown in (**k-n**) fitted with the Boltzmann function, which yielded the following results: for mTRPV1-WT (*black*), V_1/2_ = 80.1 ± 1.2 mV, and κ = 38.8 ± 1.2, Zg = 0.66 (n = 10); mTRPV1 +SUMO1 + Ubc9 (*blue*), V_1/2_ = 95.1 ± 1.5 mV, and κ = 36.0 ± 1.3, Zg = 0.71 (n = 8); mTRPV1-K823R (*olive*), V_1/2_ = 90.5 ± 1.8 mV, and κ = 37.1 ± 1.7, Zg = 0.69 (n = 6); and mTRPV1-K823R + SUMO1 +Ubc9 (*pink*), V_1/2_ = 94.6 ± 1.7 mV, and κ = 35.5 ± 1.5, Zg = 0.72 (n = 8). Data points are means ± s.e.m. (**p**-**s**) Representative traces of currents at -60 mV evoked by temperature jumps from room temperature (22 - 24 °C) to varying values as indicated (see insets for example) for CHO-K1 cells that expressed Flag-mTRPV1-WT (**p**), Flag-mTRPV1-WT + GFP-SUMO1 +Ubc9 (**q**), Flag-mTRPV1-K823R (**r**), and Flag-mTRPV1-K823R + GFP-SUMO1 +Ubc9 (**s**). Temperature was calibrated offline from the pipette current using the temperature dependence of electrolyte conductivity. (**t**) Arrhenius plot of steady-state currents shown in (p-s). Major component of the reflection of the channel strong temperature dependence was fitted to a linear equation. Error bar indicates standard deviation (s.d.). (**u**) Summaries of Q_10_ for evoked responses of mTRPV1-WT, mTRPV1-WT + SUMO1 + Ubc9, mTRPV1-K823R, and mTRPV1-K823R + SUMO1 + Ubc9, respectively. (**v**) Summary of T_threshold_ values. mTRPV1-WT (*black*), T_threshold_ = 40.7 ± 0.6 (n = 6); mTRPV1 + SUMO1 + Ubc9 (*blue*), T_threshold_ = 33.0 ± 0.5 (n = 7); mTRPV1-K823R (*olive*), T_threshold_ = 40.0 ± 0.5 (n = 7), and mTRPV1-K823R + SUMO1 + Ubc9 (*pink*), T_threshold_ = 40.9 ± 0.5 (n = 6). Data are means ± s.e.m. *** *P* < 0.0001 for Q_10_ mTRPV1 vs. mTRPV1+SUMO1+Ubc9; *** *P* < 0.0001 for T_threshold_ mTRPV1 vs. mTRPV1+SUMO1+Ubc9 by Student’s *t*-test. (**w**) Relative responses of mTRPV1-WT (*black*), mTRPV1 + SUMO1 +Ubc9 (*blue*), mTRPV1-K823R (*olive*), and mTRPV1-K823R + SUMO1 + Ubc9 (*pink*) to heat as determined using temperature jumps shown in (**p-q**) from one set of experiments. For each cell, the responses were normalized to the maximum response at 54 °C.

**
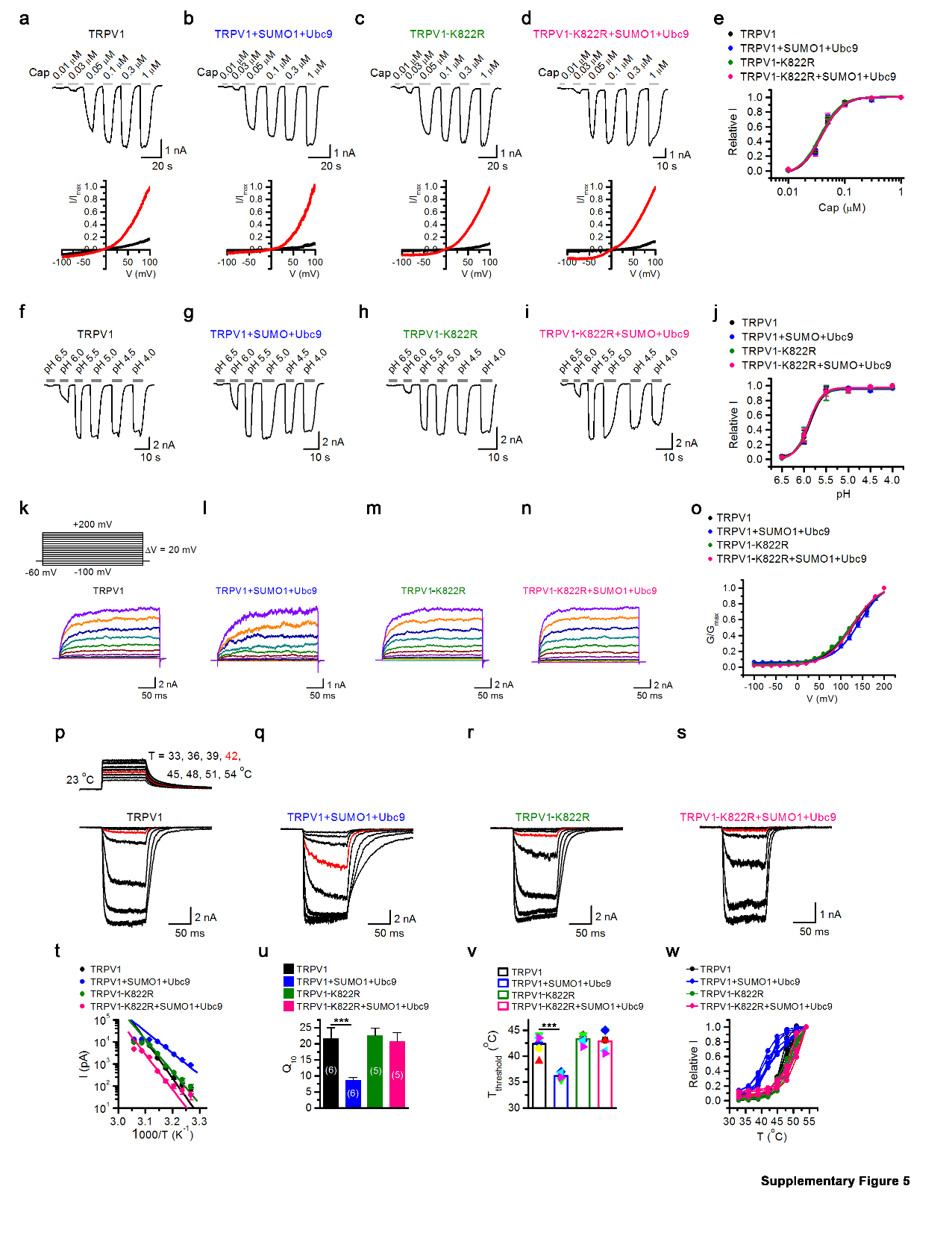
**

**Supplementary Figure 5. SUMOylation lowered the temperature threshold (T_threshold_) of rat TRPV1 activation in HEK293T cells.** (**a-d**) Upper graphs are representative traces for currents at -60 mV evoked by increasing concentrations of capsaicin as indicated for HEK293T cells that expressed Flag-rTRPV1-WT (**a**), Flag-rTRPV1-WT + GFP-SUMO1+Ubc9 (**b**), Flag-rTRPV1-K822R (**c**) and Flag-rTRPV1-K822R + GFP-SUMO1 + Ubc9 (**d**). Lower graphs are the corresponding I-V relationships of TRPV1 in response to Voltage ramp from -100 mV to +100 mV alone (*black*), or combined with 0.03 μM Cap (*red*). (**e**) Concentration-response curves for capsaicin-evoked currents. Solid lines indicate fits with the Hill equation, which yielded the following result: for rTRPV1-WT (*black*), EC_50_ = 39.1 ± 3.7 nM, and n_H_ = 2.4 ± 0.2 (n = 8); for rTRPV1-WT+SUMO1+Ubc9 (*blue*), EC_50_ = 38.0 ± 1.0 nM, and n_H_ = 2.4 ± 0.5 (n = 9); rTRPV1-K822R (*olive*), EC_50_ = 36.7 ± 3.5 nM, and n_H_ = 2.4 ± 0.4 (n = 5); for rTRPV1-K822R+SUMO1+Ubc9 (*pink*), EC_50_ = 39.0 ± 4.9 nM, and n_H_ = 2.4 ± 0.3 (n = 6). Data points are means ± s.e.m. (**f-i**) Representative traces for currents at -60 mV evoked by solutions with decreasing pH values as indicated for HEK293T cells that expressed Flag-rTRPV1-WT (**f**), Flag-rTRPV1-WT + GFP-SUMO1+Ubc9 (**g**), Flag-rTRPV1-K822R (**h**) and Flag-rTRPV1-K822R + GFP-SUMO1+Ubc9 (**i**). (**j**) Concentration-response curves for proton-evoked currents. Best fitting with Hill equation yielded the following results: for rTRPV1-WT (*black*), pH_0.5_ = 5.9 ± 0.2, and n_H_ = 2.9 ± 0.7 (n = 6); for rTRPV1-WT+SUMO1+Ubc9 (*blue*), pH_0.5_ = 5.9 ± 0.1, and n_H_ = 3.0 ± 0.4 (n = 7); for rTRPV1-K822R (*olive*), pH_0.5_ = 5.9 ± 0.1, and n_H_ = 2.7 ± 0.5 (n = 6); for rTRPV1-K822R+SUMO1+Ubc9 (*pink*), pH_0.5_ = 5.9 ± 0.1, and n_H_ = 3.0 ± 0.6 (n = 8). Data points are means ± s.e.m. (**k-n**) Representative traces of currents evoked by a family of voltage steps from -100 to +200 mV with 20 mV increments as shown in inset in (**k**) for HEK293T cells that expressed Flag-rTRPV1 (**k**), Flag-rTRPV1 + GFP-SUMO1 + Ubc9 (**l**), Flag-rTRPV1-K822R (**m**) and Flag-rTRPV1-K822R+ GFP-SUMO1 + Ubc9 (**n**). Holding potential was -60 mV. (**o**) Conductance-voltage (G-V) relationships derived from the experiments shown in (**k-l**) fitted with the Boltzmann function, which yielded the following results: for rTRPV1-WT (*black*), V_1/2_ = 131.2 ± 1.4, κ = 32.5 ± 1.3, Zg = 0.78 (n = 14); rTRPV1 +SUMO1 + Ubc9 (*blue*), V_1/2_ = 143.3 ± 1.5, κ = 29.5 ± 1.4, Zg = 0.86 (n = 14); rTRPV1-K822R (*olive*), V_1/2_ = 126.9 ± 1.5, κ = 34.1 ± 1.4, Zg = 0.75 (n = 13); and rTRPV1-K822R + SUMO1 +Ubc9 (*pink*), V_1/2_ = 128.6 ± 1.2, κ = 31.5 ± 1.1, Zg = 0.81 (n = 8). Data points are means ± s.e.m. (**p-q**) Representative traces of currents at -60 mV evoked by temperature jumps from room temperature (22 - 24 °C) to varying values as indicated (see inset in (**p**) for an example) for HEK293T cells that expressed Flag-rTRPV1-WT (**p**), Flag-rTRPV1-WT + GFP-SUMO1 +Ubc9 (**q**), Flag-rTRPV1-K822R (**r**), and Flag-rTRPV1-K822R + GFP-SUMO1 +Ubc9 (**s**). Temperature was calibrated offline from the pipette current using the temperature dependence of electrolyte conductivity. The red traces indicate the response at 42 °C. (**t**) Arrhenius plot of steady-state currents shown in (p-s). Major component of the reflection of the channel strong temperature dependence was fitted to a linear equation. Error bar indicates standard deviation (s.d.). (**u**) Summaries of Q_10_ for evoked responses of rTRPV1-WT, rTRPV1-WT + SUMO1 + Ubc9, rTRPV1-K822R, and rTRPV1-K822R + SUMO1 + Ubc9, respectively. (**v**) Summary of T_threshold_ values. rTRPV1-WT (*black*), T_threshold_ = 42.4 ± 0.6 (n = 7); rTRPV1 + SUMO1 + Ubc9 (*blue*), T_threshold_ = 36.2 ± 0.3 (n = 6); rTRPV1-K822R (*olive*), T_threshold_ = 43.3 ± 0.4 (n = 7), and rTRPV1-K822R + SUMO1 + Ubc9 (*pink*), T_threshold_ = 42.9 ± 0.8 (n = 6). Data are means ± s.e.m. *** *P* < 0.0001 for Q_10_ rTRPV1 vs. rTRPV1+SUMO1+Ubc9; *** *P* < 0.0001 for Tthreshold rTRPV1 vs. rTRPV1+SUMO1+Ubc9 by Student’s *t*-test. (**w**) Relative responses of rTRPV1-WT (*black*), rTRPV1 + SUMO1 +Ubc9 (*blue*), rTRPV1-K822R (*olive*), and rTRPV1-K822R + SUMO1 + Ubc9 (*pink*) to heat as determined using temperature jumps shown in (**p-q**) from one set of experiments. For each cell, the responses were normalized to the maximum response at 54 °C.


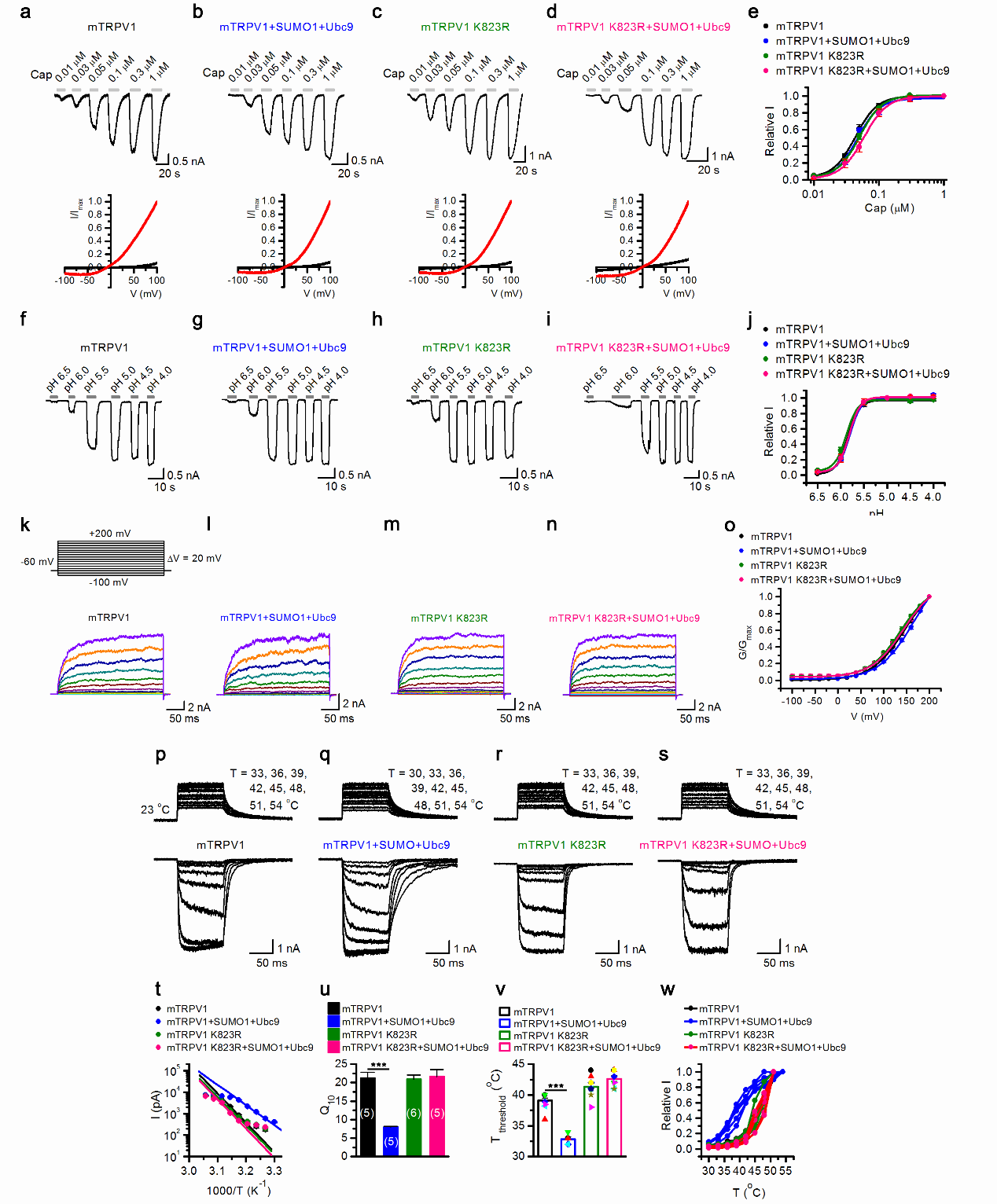


**Supplementary Figure 6. SUMOylation lowered the temperature threshold (T_threshold_) of mouse TRPV1 activation in HEK293T cells.** (**a-d**) Upper graphs are representative traces for currents at -60 mV evoked by increasing concentrations of capsaicin as indicated for HEK293T cells that expressed Flag-mTRPV1-WT (**a**), Flag-mTRPV1-WT+GFP-SUMO1+Ubc9 (**b**), Flag-mTRPV1-K823R (**c**) and Flag-mTRPV1-K823R + GFP-SUMO1 + Ubc9 (**d**). Lower graphs are the corresponding I-V relationships of TRPV1 in response to Voltage ramp from -100 mV to +100 mV alone (*black*), or combined with 0.03 μM Cap (*red*). (**e**) Concentration-response curves for capsaicin-evoked currents. Solid lines indicate fits with the Hill equation, which yielded the following result: for mTRPV1-WT (*black*), EC_50_ = 43.3 ± 1.3 nM, and n_H_ = 2.6 ± 0.2 (n = 11); for mTRPV1-WT+SUMO1+Ubc9 (*blue*), EC_50_ = 46.0 ± 5.9 nM, and n_H_ = 2.5 ± 0.8 (n = 8); mTRPV1-K823R (*olive*), EC_50_ = 50.4 ± 1.2 nM, and n_H_ = 2.5 ± 0.1 (n = 11); for mTRPV1-K823R+SUMO1+Ubc9 (*pink*), EC_50_ = 58.4 ± 2.9 nM, and n_H_ = 2.4 ± 0.3 (n = 6). Data points are means ± s.e.m. (**f-g**) Representative traces for currents at -60 mV evoked by solutions with decreasing pH values as indicated for HEK293T cells that expressed Flag-mTRPV1-WT (**f**), Flag-mTRPV1-WT+GFP-SUMO1+Ubc9 (**g**), Flag-mTRPV1-K823R (**h**) and Flag-mTRPV1-K823R + GFP-SUMO1+Ubc9 (**i**). (**j**) Concentration-response curves for proton-evoked currents. Best fitting with Hill equation yielded the following results: for mTRPV1-WT (*black*), pH_0.5_ = 5.9 ± 0.1, and n_H_ = 3.5 ± 0.7 (n = 11); for mTRPV1-WT+SUMO1+Ubc9 (*blue*), pH_0.5_ = 5.8 ± 0.1, and n_H_ = 3.5 ± 0.7 (n = 10); for mTRPV1-K823R (*olive*), pH_0.5_ = 5.9 ± 0.1, and n_H_ = 3.5 ± 0.6 (n = 7); for mTRPV1-K823R+SUMO1+Ubc9 (*pink*), pH_0.5_ = 5.8 ± 0.1, and n_H_ = 3.6 ± 0.2 (n = 7). Data points are means ± s.e.m. (**k-n**) Representative traces of currents evoked by a family of voltage steps from -100 to +200 mV with 20 mV increments as shown in inset in (**k**) for HEK293T cells that expressed Flag-mTRPV1 (**k**), Flag-mTRPV1 + GFP-SUMO1 + Ubc9 (**l**), Flag-mTRPV1-K823R (**m**) and Flag-mTRPV1-K823R+ GFP-SUMO1 + Ubc9 (**n**). Holding potential was -60 mV. (**o**) Conductance-voltage (G-V) relationships derived from the experiments shown in (**p-s**) fitted with the Boltzmann function, which yielded the following results: for mTRPV1-WT (*black*), V_1/2_ = 147.1 ± 2.2 mV, and κ = 36.5 ± 1.2, Zg = 0.70 (n = 15); mTRPV1 +SUMO1 + Ubc9 (*blue*), V_1/2_ = 168.0 ± 3.5 mV, and κ = 38.5 ± 1.4, Zg = 0.66 (n = 15); mTRPV1-K823R (*olive*), V_1/2_ = 136.7 ± 1.8 mV, and κ = 33.6 ± 1.1, Zg = 0.76 (n = 9); and mTRPV1-K823R + SUMO1 +Ubc9 (*pink*), V_1/2_ = 144.0 ± 2.4 mV, and κ = 35.3 ± 1.4, Zg = 0.72 (n = 7). Data points are means ± s.e.m. (**p-s**) Representative traces of currents at -60 mV evoked by temperature jumps from room temperature (22 - 24 °C) to varying values as indicated (see inset for example) for HEK293T cells that expressed Flag-mTRPV1-WT (**p**), Flag-mTRPV1-WT + GFP-SUMO1 +Ubc9 (**q**), Flag-mTRPV1-K823R (**r**), and Flag-mTRPV1-K823R + GFP-SUMO1 +Ubc9 (**s**). Temperature was calibrated offline from the pipette current using the temperature dependence of electrolyte conductivity. (**t**) Arrhenius plot of steady-state currents shown in (p-s). Major component of the reflection of the channel strong temperature dependence was fitted to a linear equation. Error bar indicates standard deviation (s.d.). (**u**) Summaries of Q_10_ for evoked responses of mTRPV1-WT, mTRPV1-WT + SUMO1 + Ubc9, mTRPV1-K823R, and mTRPV1-K823R + SUMO1 + Ubc9, respectively. (**v**) Summary of T_threshold_ values. mTRPV1-WT (*black*), T_threshold_ = 39.1 ± 0.4 (n = 6); mTRPV1 + SUMO1 + Ubc9 (*blue*), T_threshold_ = 32.8 ± 0.4 (n = 5); mTRPV1-K823R (*olive*), T_threshold_ = 41.3 ± 0.6 (n = 9), and mTRPV1-K823R + SUMO1 + Ubc9 (*pink*), T_threshold_ = 42.6 ± 0.6 (n = 8). Data are means ± s.e.m. *** *P* < 0.0001 for Q_10_ mTRPV1 vs. mTRPV1+SUMO1+Ubc9; *** *P* < 0.0001 for T_threshold_ mTRPV1 vs. mTRPV1+SUMO1+Ubc9 by Student’s *t*-test. (**w**) Relative responses of mTRPV1-WT (*black*), mTRPV1 + SUMO1 +Ubc9 (*blue*), mTRPV1-K823R (*olive*), and mTRPV1-K823R + SUMO1 + Ubc9 (*pink*) to heat as determined using temperature jumps shown in (**p-q**) from one set of experiments. For each cell, the responses were normalized to the maximum response at 54 °C.


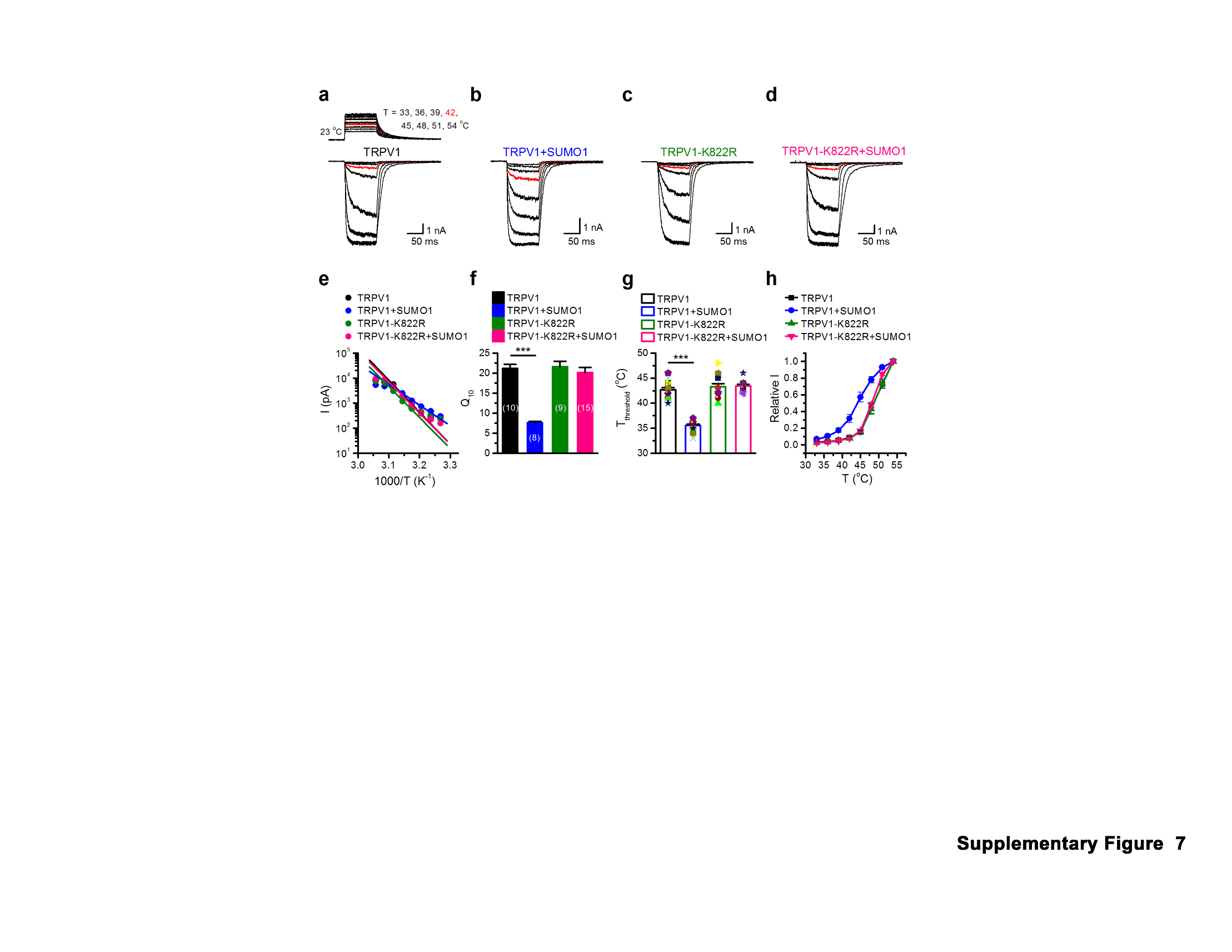


**Supplementary Figure 7. SUMOylation** **lowered the temperature threshold (T_m_) of rat TRPV1 activation.** (**a-d**) Representative responses to a family of temperature jumps ranging from 33-54 °C (see inset in (**a**) for an example) for HEK293T cells that expressed TRPV1 (**a**), TRPV1+SUMO1 (**b**), TRPV1-K822R (**c**), and TRPV1 K822R+SUMO1 (**d**). Temperature was calibrated offline from the pipette current using the temperature dependence of electrolyte conductivity. The red traces indicate the response at 42 ^o^C. The holding potential was -60 mV. (e) Arrhenius plots of current responses for determination of temperature dependence. Major component of the reflection of the channel strong temperature dependence was fitted to a linear equation. Error bar indicates standard deviation (s.d.). (f-g) Comparison of Q_10_ derived from the linear fits in (e) and T_threshold_ changes. The values of Q_10_ and T_threshold_ are as following, Q_10_=21.2 ± 1.1 (n=10) and T_threshold_= 42.7 ± 0.4 (n=11) for TRPV1, Q_10_=7.6 ± 0.4 (n=8) and T_threshold_= 35.5 ± 0.3 (n=9) for TRPV1+SUMO1, Q_10_=21.6 ± 1.3 (n=9) and T_threshold_=43.3 ± 0.6 (n=9) for TRPV1-K822R, Q10=20.2 ± 1.2 (n=15) and T_threshold_=43.4 ± 0.3 (n=13) for TRPV1-K822R+SUMO1. Data are means ± s.e.m. *** *P* < 0.0001, TRPV1 vs. TRPV1+SUMO1 by Student’s *t*-test. (h) Comparison of temperature-dependent responsive curves. Relative responses of TRPV1 (*black*), TRPV1+SUMO1 (*blue*), TRPV1 K822R (*olive*), and TRPV1 K822R+SUMO1 (*pink*) to heat are normalized to each maximum response at 54 °C.


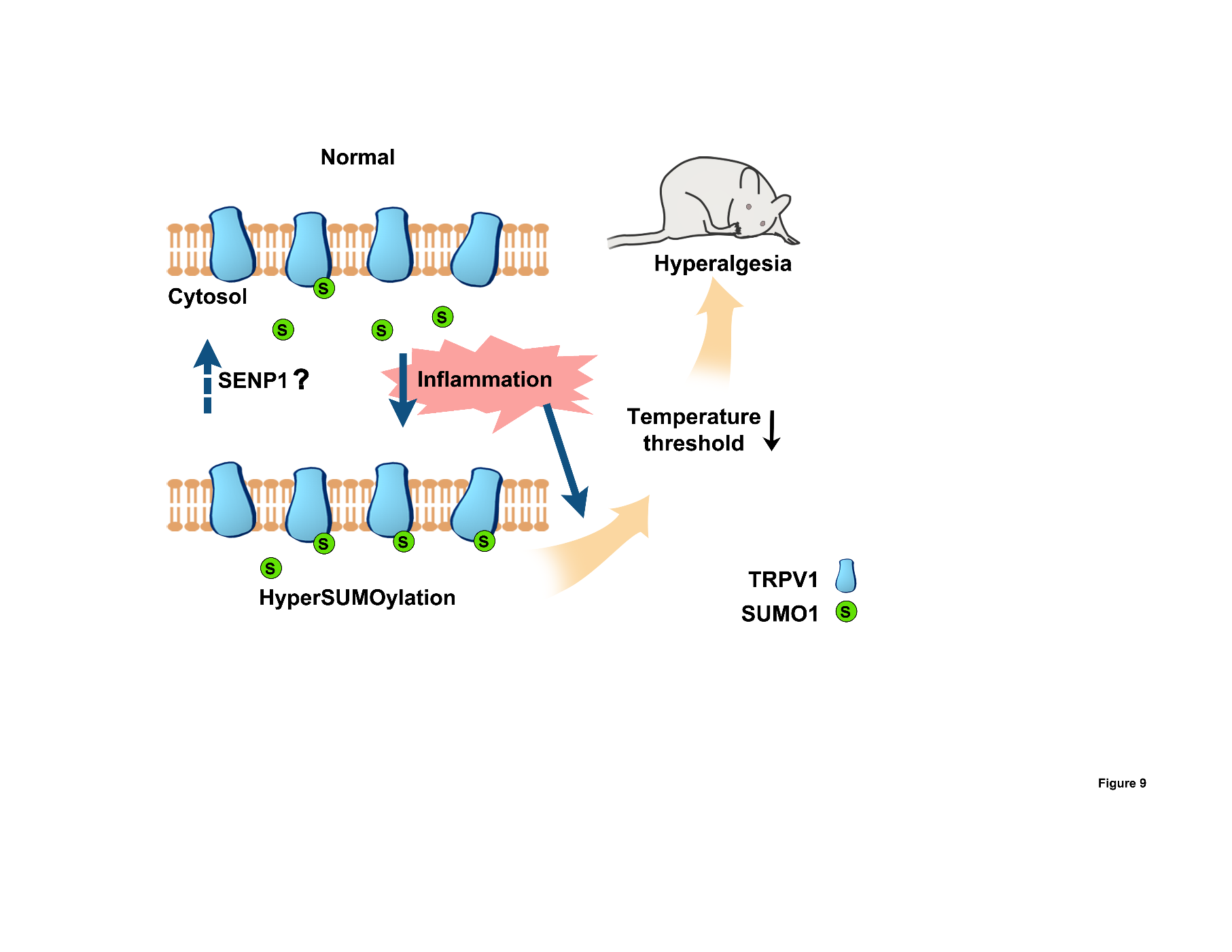


**Supplementary Figure 8. Schematic model of TRPV1 SUMOylation in the regulation of inflammatory thermal hyperalgesia.** The SUMOylation/deSUMOylation status of TRPV1 is regulated by peripheral inflammation and SENP1. SUMOylation lowers the temperature threshold for TRPV1 channel activation, leading to inflammatory thermal hyperalgesia. Other constituents of inflammation may facilitate this process.


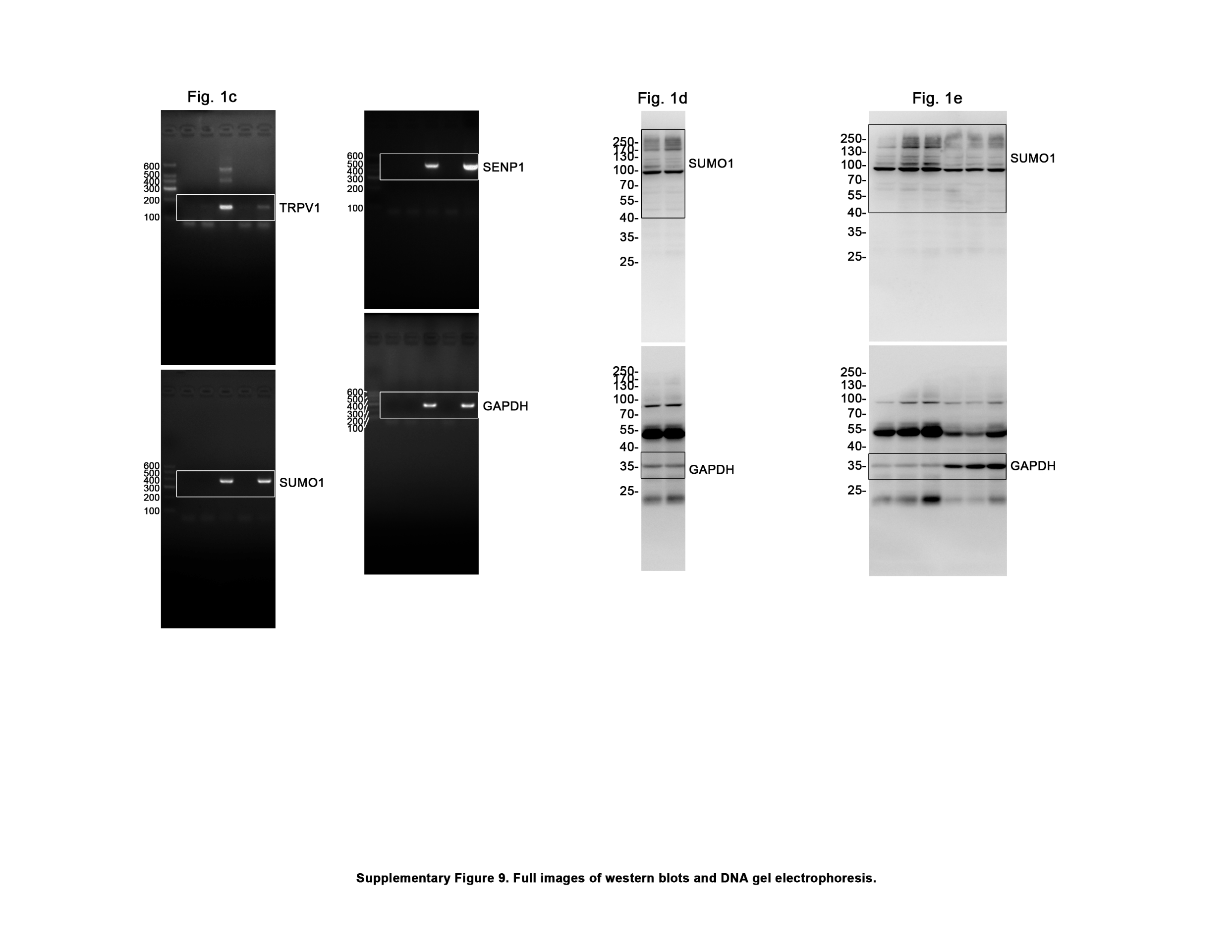


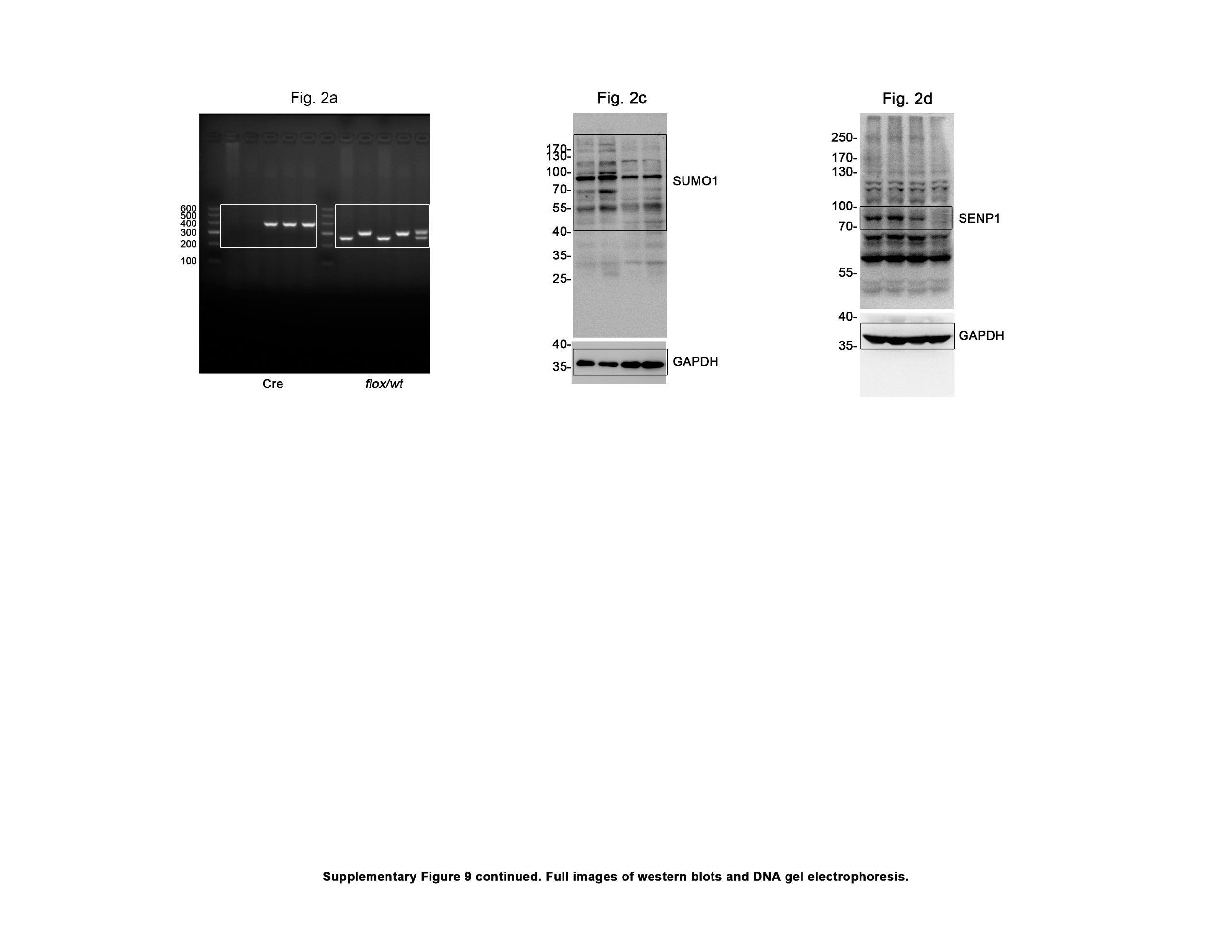


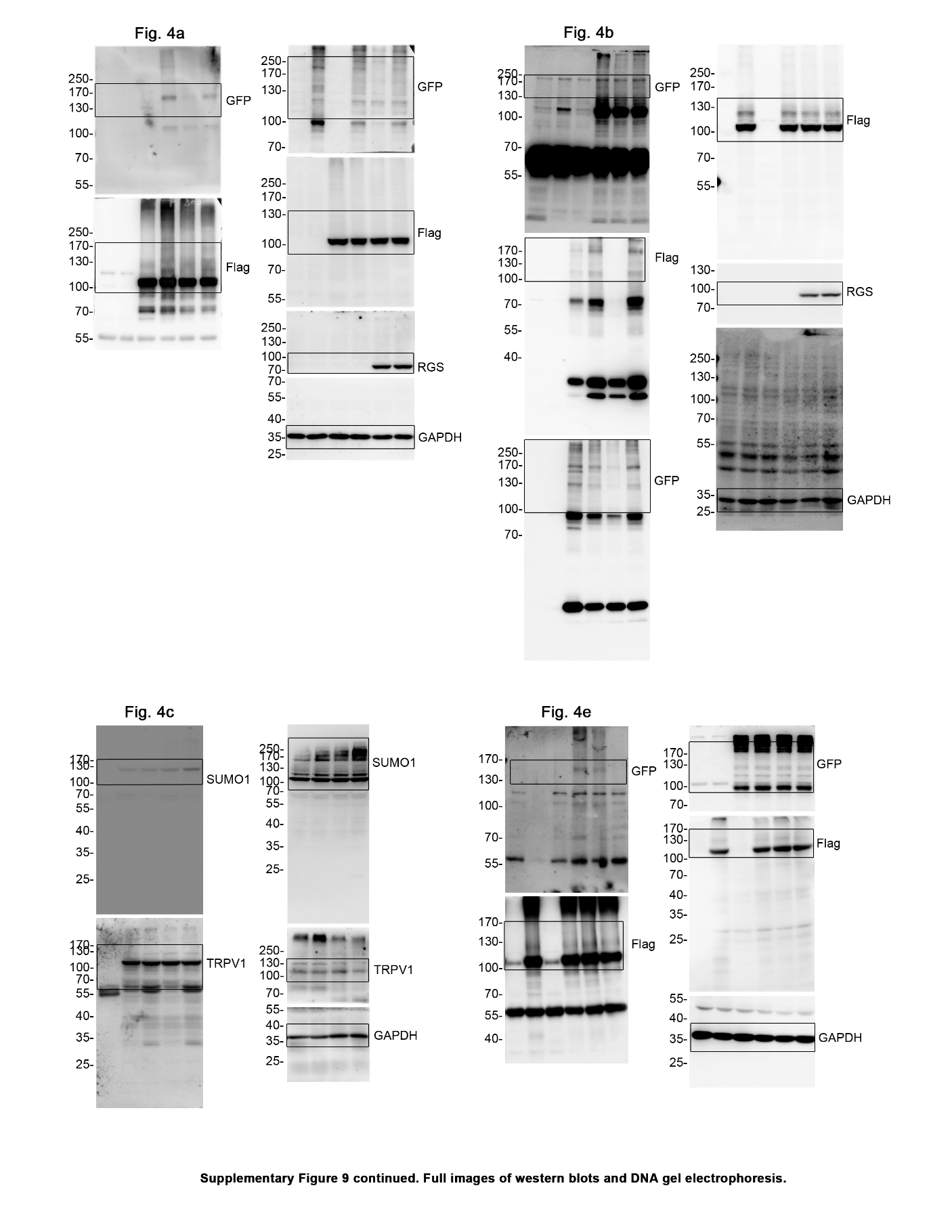

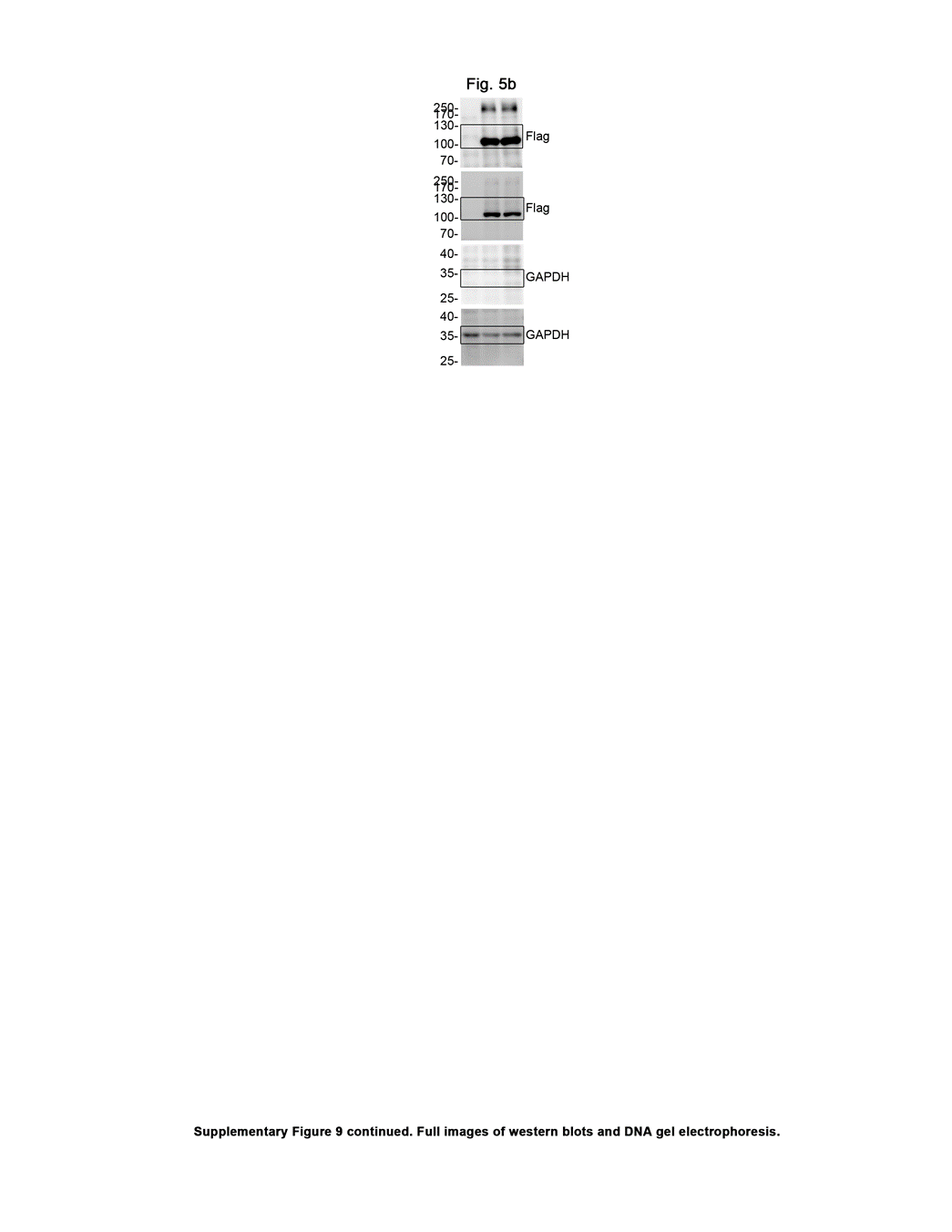


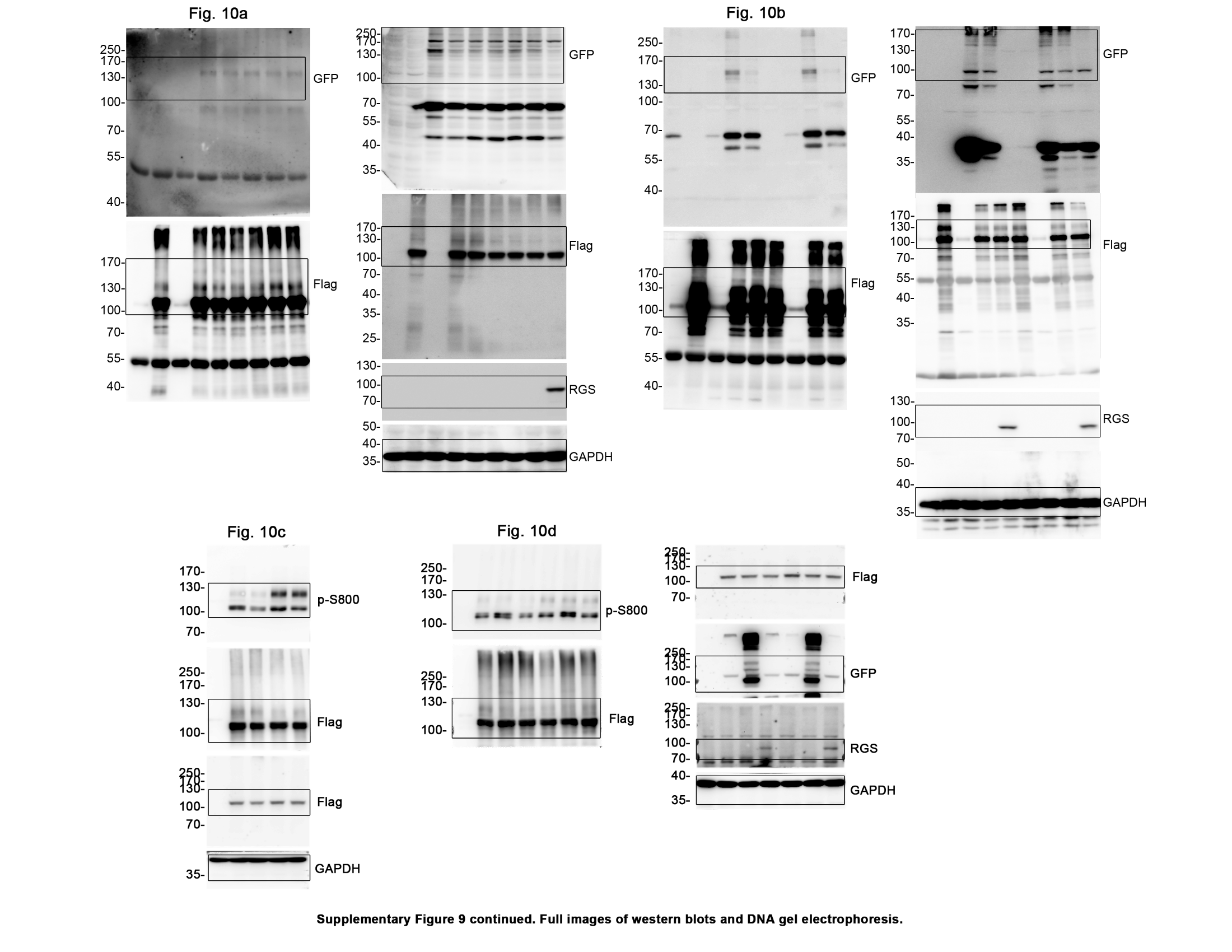


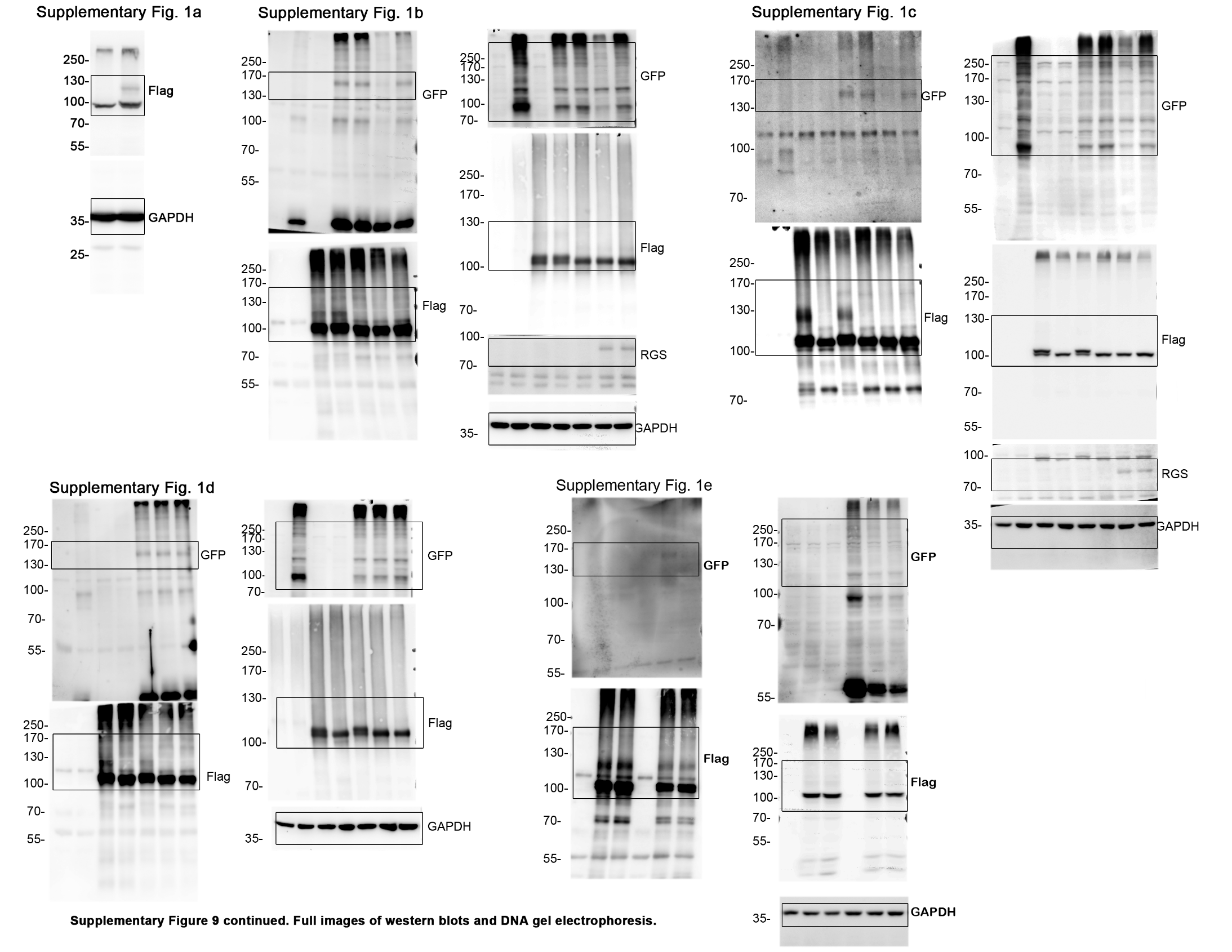





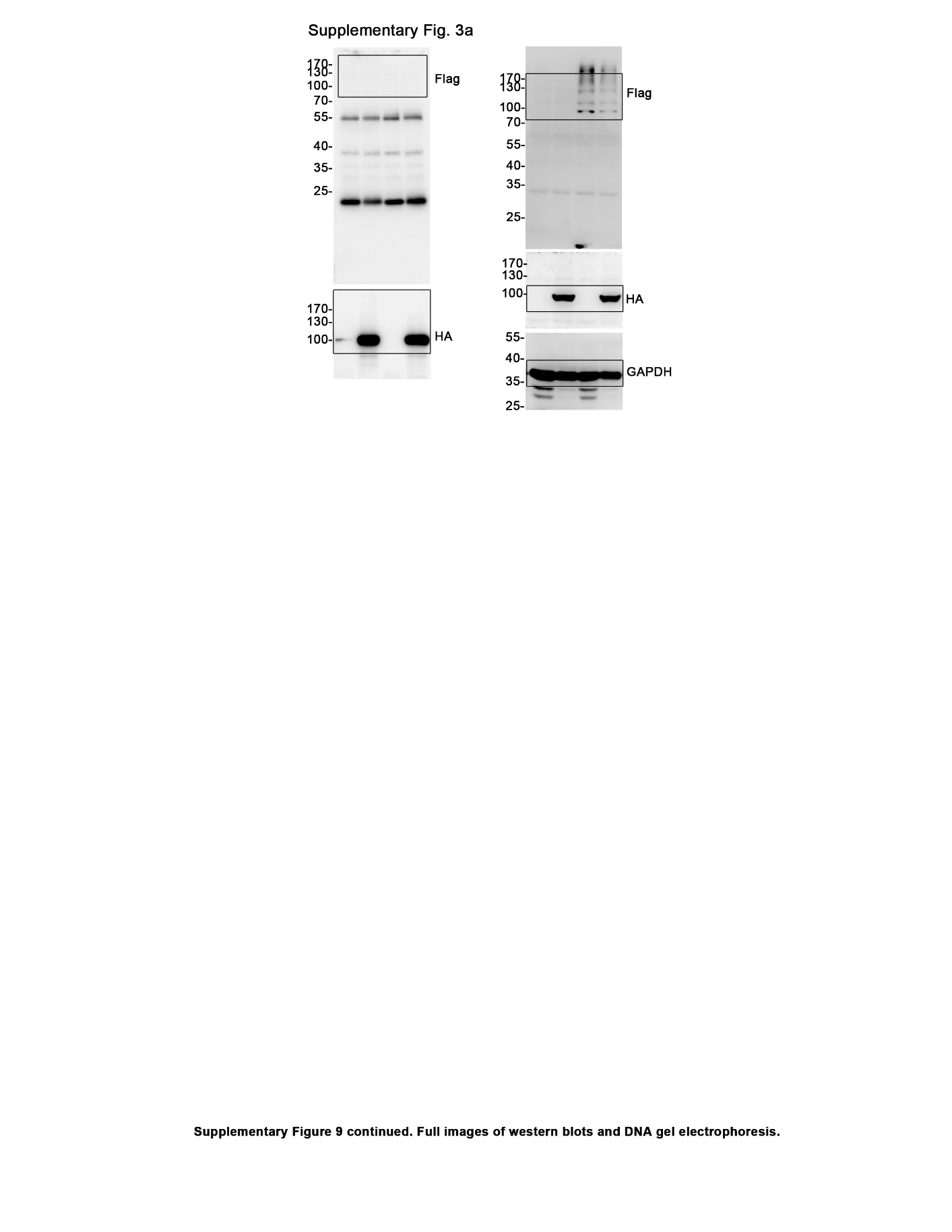

Supplement: Supplementary file 1 — Supplementary information [file 41467_2018_3974_MOESM1_ESM.docx]
